# Organotypic cultures as aging associated disease models

**DOI:** 10.18632/aging.204361

**Published:** 2022-11-22

**Authors:** Martina M. Sanchez, Isabella A. Bagdasarian, William Darch, Joshua T. Morgan

**Affiliations:** 1 Department of Bioengineering, University of California, Riverside, CA 92521, USA

**Keywords:** tissue engineering, organotypic, skeletal muscle, skin, intestine

## Abstract

Aging remains a primary risk factor for a host of diseases, including leading causes of death. Aging and associated diseases are inherently multifactorial, with numerous contributing factors and phenotypes at the molecular, cellular, tissue, and organismal scales. Despite the complexity of aging phenomena, models currently used in aging research possess limitations. Frequently used *in vivo* models often have important physiological differences, age at different rates, or are genetically engineered to match late disease phenotypes rather than early causes. Conversely, routinely used *in vitro* models lack the complex tissue-scale and systemic cues that are disrupted in aging. To fill in gaps between *in vivo* and traditional *in vitro* models, researchers have increasingly been turning to organotypic models, which provide increased physiological relevance with the accessibility and control of *in vitro* context. While powerful tools, the development of these models is a field of its own, and many aging researchers may be unaware of recent progress in organotypic models, or hesitant to include these models in their own work. In this review, we describe recent progress in tissue engineering applied to organotypic models, highlighting examples explicitly linked to aging and associated disease, as well as examples of models that are relevant to aging. We specifically highlight progress made in skin, gut, and skeletal muscle, and describe how recently demonstrated models have been used for aging studies or similar phenotypes. Throughout, this review emphasizes the accessibility of these models and aims to provide a resource for researchers seeking to leverage these powerful tools.

## INTRODUCTION

Chronic aging associated disease (AAD) remains one of the defining medical challenges of our time, representing 95% of direct health costs for seniors and driving expected Medicare spending to over $1.2 trillion by 2024 [1, 2]. Further, patient care is complicated by the convolution of systemic factors, multiple diseases, and conflicting treatment plans. Indeed, patients co-presenting two or more AADs are common and costly, with patients managing 2 or more chronic conditions representing over 70% of healthcare spending [3]. This complexity is reflected at the molecular level, with numerous mechanisms implicated in the aging process. These mechanisms prominently include inflammation, oxidation, metabolic and mitochondrial dysfunction, telomere shortening, and cellular senescence; we direct readers to other reviews on the molecular drivers of aging [4, 5]. Despite strong research efforts, connecting the host of molecular changes to development of effective treatments for AAD remains challenging. Identifying and intervening in early stages of chronic disease remains difficult with the slow degeneration distributed over years, evaluation of molecular markers occurring long after pathogenesis, and convolution of many subtle pathway dysregulations. A major contributor to these challenges is the limitations of commonly used *in vivo* and *in vitro* models.

Animal models of aging broadly follow the phenotypes of human aging and can be used to model specific AAD [6]. However, specific mechanisms (e.g., immune function or telomere regulation) differ in important ways [7]. Further, many human AAD lack analogs in naturally occurring animal disease, especially in more cost-effective rodent models. Prime examples of this are cardiovascular disease [8], primary open angle glaucoma [9–11], and neurodegeneration [12]. While animal studies will remain an essential component of biomedical research for the foreseeable future, there is longstanding recognition of their limitations [13] and consideration of reduction strategies [14, 15].

Similarly, conventional two-dimensional *in vitro* culture has been indispensable in understanding the molecular mechanisms associated with aging [16]; advantages include cost-effectiveness, replicability, ease of chemical and genetic manipulation, and accessibility to analytical and imaging methods [17, 18]. Unfortunately, these advantages come with a number of known limitations including modified sensitivity to pharmacological agents, distorted expression profiles, abnormal morphology, and altered differentiation schema [7, 19, 20]. To address these limitations in both conventional *in vitro* and *in vivo* animal models, there has been increasing development of more physiologically representative *in vitro* models. Ideally, these models incorporate human cells and more accurately reflect the mechanical, physicochemical, biochemical, and cellular context of *in vivo* tissue. Models that mimic the heterogeneous cell composition and organization of native tissue are generally referred to as organotypic, a category that includes both *ex vivo* and *in vitro* models. Key examples of *in vitro* organotypic models include organoid, organ-on-a-chip, organotypic tissue slice, and tissue engineered organotypic models.

Organoid models are generated by a number of different source materials including tissue fragments and explants, reconstituted primary cells, and stem cells [7, 18]. While there is no single definition of organoid models, broadly speaking, they are constructed through the self-assembly of patient, primary, or stem cells; exhibit cellular and matrix organization mimetic of the *in vivo* environment; and a heterogeneous cell population mimetic of native tissue. Organ-on-a-chip models generally possess these same advantages, with additional potential features consisting of defined structural patterning of the cells, microfluidic or environmental control of the system, and incorporation of sensors or physiological readouts [21–23]. Organotypic tissue slice cultures use thinly sliced sections of tissue, preserving the cellular microenvironment and tissue organization; these have been used in a range of tissues, including heart, lung, liver, and most prominently, brain [24–30]. These model classes have enabled significant contributions to research and drug discovery, including in the aging field. A notable example is in brain, where organoids and organotypic slices have been used to research aging associated degeneration, Alzheimer’s, dementia, and Parkinson’s; the progress in brain organotypic models has been extensively reviewed by others [31, 32]. These model classes have enabled significant contributions to research and drug discovery, yet have notable limitations. For example, organoids and organ-on-a-chip models are typically small (sub-mm) due to the lack of vasculature and diffusion limits of oxygen and metabolites [33–35], although organ-on-a-chip models sometimes address this issue through microfluidic perfusion. Further, organoid and slice models often require patient or freshly isolated animal tissue that can be difficult to acquire; organ-on-a-chip models often rely on specialized microfabrication techniques that not all aging research labs can easily implement. Another culture category and topic of this review, tissue engineered organotypic culture, leverages the progress in tissue engineering to create tissue-scale and physiologically relevant *in vitro* models.

Tissue engineering, a term first coined over three decades ago, has long held promise for the *in vitro* creation of fully functional tissue grafts [36, 37], however, numerous challenges have limited development. *In vitro* development of skin grafts, one of the initial targets of the field [37], is only just now entering medical use as an adjunct to traditional therapy [38], with fully functional engineered skin still unavailable [39]. This is broadly representative of the current state of the field, which, despite significant research progress, have demonstrated limited clinical application of grafts. However, for the past two decades, researchers have repurposed engineered tissues towards research questions [14, 40–42]. Similar to organoid and organ-on-a-chip cultures, these models are constructed from organotypic cell populations, but typically offer a greater degree of control over the tissue architecture and included cell populations. Cells and structures can be patterned or allowed to self-assemble depending on the needs of the research [43, 44]; similarly, cell populations and sub-populations can be easily controlled or replaced to reflect tissue health and disease. Leaders in tissue engineering have urged the simplicity and cost-effectiveness of design [34, 45], and this is reflected into the increasing number of methods papers and decreasing costs of biomaterials [14, 40]. These models represent a powerful and accessible set of tools for aging research; and are likely to become increasingly relevant as the field moves towards bridging cellular and tissue-scale hallmarks of aging.

In this review, we summarize research efforts and potential for utilizing organotypic and tissue engineered models for aging and AAD. To streamline the review, it is broken into independent sections for skin, intestine, and skeletal muscle; which represent well-developed fields and are important tissues in physiological aging and AAD. Each section briefly covers important facets of the aging physiology in the tissue system, before describing current and emerging organotypic techniques and their application to aging. In each tissue section, we describe the advantages (and limitations) of organotypic models in elucidating aging mechanisms at the cellular and tissue scales, as well as highlighting the key methodological and accessibility factors.

## Demonstrative organotypic models relevant to aging tissue

### Skin

#### Native skin aging

**Figure 1:**
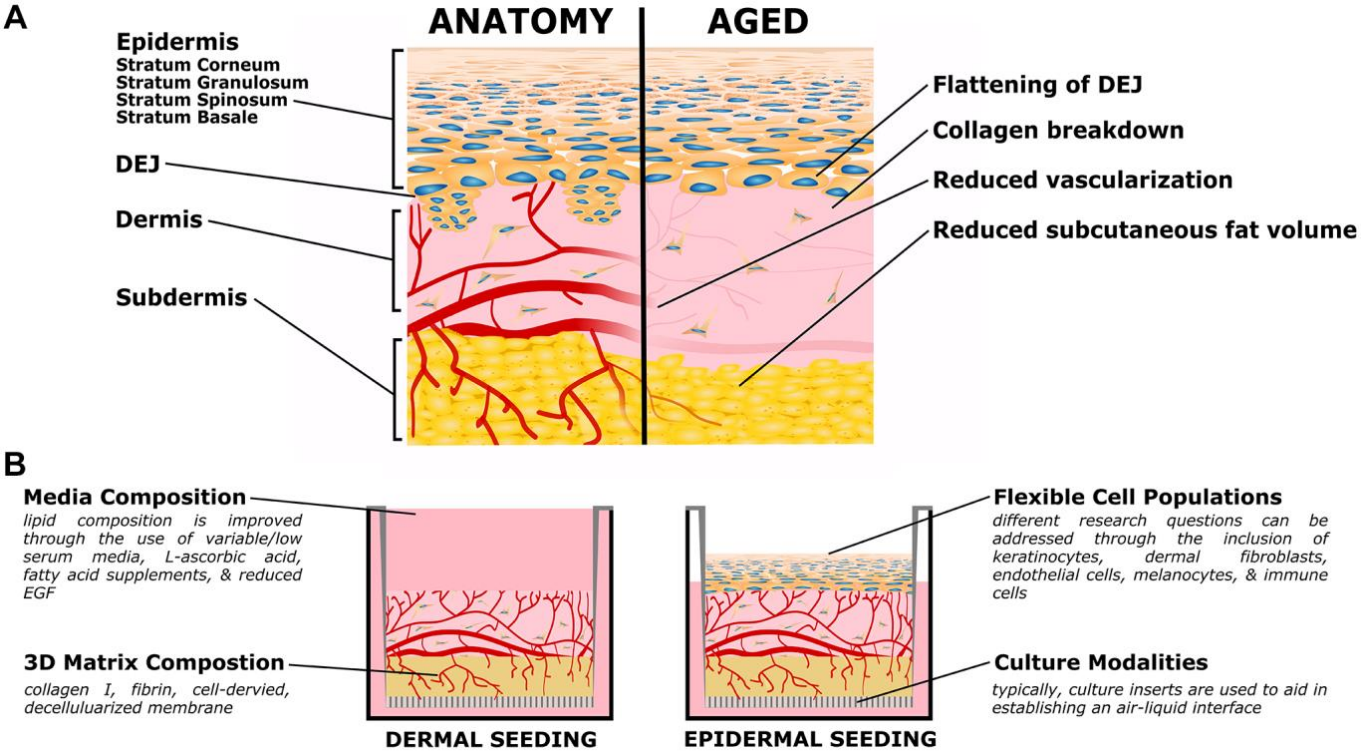
**Organotypic models of skin aging.** (**A**) Simplified skin anatomy and aging phenotypes. Skin can be separated into epidermal, dermal, and hypodermal layers. The epidermis is composed of Stratum Basale, Spinosum, Granulosum, and Corneum, composed of increasingly differentiated epidermal cells. The dermal-epidermal junction (DEJ) connects the basement membrane of the Stratum Basale to the upper (papillary) dermis, and is characterized by small dermal extensions (or papilla) into the epidermis. The DEJ flattens with age. The dermis is a collagen rich tissue supported by dermal fibroblasts. The subdermis (or hypodermis) is an important adipose compartment that contributes to overall metabolic function; this tends to thin with age. Both the dermis and subdermis are highly vascularized, important for thermal regulation; in age vascularization is reduced. The above schematic is simplified to focus on the level of current organotypic models, nerves, melanocytes, immune cells, and other components of *in vivo* skin are not pictured. (**B**) Organotypic skin models, also referred to as Human Skin Equivalents (HSE), typically consist of a dermal/subdermal culture grown on a permeable culture support (left), followed by seeding and differentiation of epidermis at the air-liquid interface (ALI). Benefits of this style is the accessibility of the culture format, ready customization of the specific cell populations (both immortalized or primary, patient specific, or transgenic disease models), and customization of the matrix and media formulations.

Skin is one of the largest organs of the body and has functional roles in immune response, physical protection, and thermal regulation [46]. A simplification of skin anatomy is shown in Figure 1A. As aging occurs, skin function and healing capacity is reduced, with key aging changes summarized in Table 1. Skin aging is frequently divided into two related processes: intrinsic and extrinsic aging [47–50]. Intrinsic aging, also referred to as chronological aging, includes genetic and hormonal changes and the progression from cell maturity to cellular senescence [47, 50]. Extrinsic aging, also referred to as environmental aging, represents the impact of the environment, including: photoaging associated with sun exposure [47, 51, 52], cigarette smoking, pollution, chemical exposure, and trauma [50]. Due to the different underlying mechanisms, characteristics of each type of aged skin are different. Chronologically (intrinsically) aged skin presents as unblemished, smooth, pale, dry, lower elasticity, and has fine wrinkles while environmentally (extrinsically) aged skin has coarse wrinkling, rough textures, pigmentation changes, and lower elasticity [50, 53].

**Table 1:** Prominent phenotypes of aging skin.

**Prominent Aging Phenotypes**	**References**
Lower elasticity, increased fragility, and wrinkle formation	[47, 50, 53, 54]
Increased collagen disorganization, accumulation of advanced glycation end products, and changes in (GAG) and (PG) concentrations/organization	[49, 53, 55–61]
Flattening of the dermal epidermal junction	[50, 52]
Decreased dermal vasculature	[62]
Reduced subcutaneous fat volume	[50]
Increased cellular senescence	[49, 63]
Decreased cell population and turnover, including melanocytes, epidermal cells, dermal fibroblasts, and immune cells	[50, 63, 64]
Reduced barrier function coupled with changes in the stratum corneum, lipid composition, and filaggrin expression	[65–69]

Structural changes in intrinsically aged skin include decreased dermal vasculature [62]; changes in dermal elasticity and increased collagen disorganization [70, 71]; build-up of advanced glycation end products (AGEs) and changes in glycosaminoglycan (GAG) and proteoglycan (PG) concentrations/organization contributing to stiffening of dermal structure and frailty, and decreased hydration [49, 53, 55–61]; imbalance of tissue inhibitors and matrix metalloproteinases (MMPs) resulting in imbalance between collagen deposition and breakdown [50, 72]; and flattening of the dermal epidermal junction/loss of rete ridges [50, 52, 63, 64, 73]. Aging also contributes to variations in epidermal and dermal thickness [63, 64, 74, 75] and reduced subcutaneous fat volume [50]. There are also many changes related to cell population in all three main skin compartments (epidermal, dermal, hypodermal) including reduced epidermal cell turnover [50, 73], drop in number of active melanocytes [50]; decreases in dermal fibroblast concentrations [64], decreases in immune cells [63, 64] and immune function. Abnormalities of skin barrier (a major function of the epidermis) occur during aging and often present as dryness or skin irritation. In aged skin, barrier function has been studied in the context of decreases of filaggrin [65], increases in pH (5 to ~5.6), altered lipid presence [66, 67], and changes in cornified envelope arrangement [63, 68, 69, 76]. These changes add to fragility of older skin and increase chances of infection [54], it remains unclear exactly how these changes take place and what mechanisms are controlling them.

On the molecular scale, expression levels of soluble factors, proteins, and vitamins are both effects and contributors to aging phenotypes. Examples include upregulation of stress regulatory proteins (hypoxia-inducible factors, nuclear factor kappa-light chain-enhancer) [63], increases in AP-1 (leading to increased collagen breakdown via MMP activity) [52, 72], and declines in vitamin D production by the epidermis [63]. These changes are largely attributed to increases in reactive oxygen species (ROS) [52, 63], DNA mutations (including mitochondrial DNA), telomere shortening [63], increased cell senescence, and hormonal changes [49, 63]. Changes in skin aging have been associated with fluctuations in expression patterns of integrins including α6 and ß1 integrins [57, 59, 71, 77, 78]. In healthy human skin, α6 and ß1 (and other α/ß subunits) integrin expression are localized on the basal side of basal keratinocytes [57, 78]. Defects in integrin expression are present in human blistering skin diseases with supporting evidence in knockout mice [78] and also in aged human skin [57, 59], although further work is necessary to understand how integrin expression changes in aging.

Aging in the skin has sex-related differences as well, specifically, sex is linked to faster thinning of the dermis and collagen density decline in males as opposed to females [50, 79]. Males undergo a decline in androgen levels while estradiol levels are constant, these changes result in a linear decline of skin thickness and collagen content in men [70]. Women experience both androgen and estrogen decline linearly and an additional post-menopausal estrogen decline which is linked to lower collagen content, lower skin moisture and capacity to hold water, lessened wound healing response, thinner skin, and lower skin elasticity [50, 53, 70, 80]. Detailed summary and discussion of sex-related changes in skin aging have been previously reviewed [70].

These intrinsic mechanisms are compounded by environmental skin aging (extrinsic aging) [49, 52, 63]. A key example is the effects of ultraviolet (UV) irradiation (an extrinsic aging mechanism), which accelerates telomere shortening and DNA damage present with intrinsic aging [50, 81]. Other extrinsic aging and examples of compounding UV effects are discussed in previous literature [49, 71, 82–88]. Overall, skin aging at the molecular, cellular, and tissue levels continues to be a field of active research. While *in vivo* and traditional cell culture models remain important tools, there is increasing interest in more physiologically relevant culture models, and there is a growth in recent studies employing organotypic skin models (OSCs).

#### Tissue engineered skin models

Researchers have used organotypic models to study skin biology since the 1980s [89, 90], and the methodology are increasingly accessible. OSCs are also commonly referred to as human skin equivalents (HSEs) or full-thickness skin models; they typically have dermal and properly stratified epidermal layers (Figure 1B). These models have proven useful for studying skin development, evaluating cytotoxicity, studying wound healing, and more recently as disease and aging models. OSCs are highly customizable and allow for control of organotypic cell populations, genotypes, and culture conditions to enable carefully controlled studies on tissue-level biology. OSCs have the capacity to be used for in depth aging studies without the dangers of human trials or expensive animal models; with long-term culture stability for chronic studies (typical culture lengths of 8–12 weeks) [91–93]. Most commonly, OSCs contain dermal fibroblasts and keratinocytes and are cultured at an air-liquid interface for epidermal differentiation and stratification. However, with the growth of interest in heterogeneous cell-cell communication, an increasing number of models have been demonstrated with additional cell populations [71, 94, 95]. These include vascular endothelial cells [92, 93, 96–101], immune cells [102–105], adipose derived stem cells and adipocytes from adipose derived stem cells [106–108], embryonic stem cells [71], melanocytes [109–111] and melanocytes derived from induced pluripotent stem cells [112]. With this customizability and a growing number of accessible protocols, OSCs represent a useful tool for studying skin aging; exemplar applications are discussed below, first for disease generally and then with aging specifically.

OSCs have been used in a number of disease studies, both directly and as “hybrid” studies where a humanized OSC is grafted onto immunodeficient mice. Additionally, models have been shown useful for testing potential therapeutic techniques for debilitating skin disorders or injuries [113]. OSC skin disorder models include: psoriasis [114–116], recessive dystrophic epidermolysis bullosa [117, 118], lamellar ichthyosis [119], Netherton syndrome [120], congenital pachyonychia [121], Junctional epidermolysis bullosa [71, 122], and fibrosis [123–125]. Of these disease models, the fibrosis model by Varkey et al. is especially interesting for its potential to be adapted to use as an aging model. In this study, OSCs were generated using either deep dermal fibroblasts or superficial dermal fibroblasts in combination with normal human keratinocytes [123]. They found that the antifibrotic properties of deep dermal fibroblasts and the fibrotic properties of superficial fibroblasts influence OSC characteristics. Authors found that when compared to constructs with superficial or mixed fibroblast populations, OSCs with deep fibroblasts had higher levels of IL-6, reduced TGF-β1 production, higher PDGF expression, and epidermal formation was less defined and less continuous [123]. This model is potentially interesting as a platform for aging research, as TGF-β is implicated in skin aging through regulation of matrix metalloprotease activity [126, 127]. The work of Varkey et al. highlights the usefulness of OSCs to study signaling between specific cellular subpopulations in a controlled way; this approach could be readily adapted to aging studies. Given this potential, it is unsurprising that several research groups have used OSCs in aging research, which we highlight in the next section.

#### Tissue engineered skin models to study aging

As OSCs are stable for long culture periods (>17 weeks), using the extended culture time to study intrinsic aging is perhaps one of the most straightforward techniques and can be combined with other aging models and/or cell types [73]. With this model, authors demonstrated that extended culture (using a non-traditional matrix of collagen-glycosaminoglycan-chitosan porous polymer) exhibited several age-related aspects similar to those that occur with *in vivo* aging, including decreases in epidermal thickness, decreases in hyaluronan expression, increases of the aging biomarker p16^Ink4a^, decreases in keratinocyte proliferation over time, loss of expression of healthy epidermal markers, and basement membrane alterations. Another straightforward application of OSCs in aging is studying the impact of senescent cells. A number of studies have incorporated senescent fibroblasts into OSCs to generate models that recapitulate many of the features of *in vivo* aged skin. [74, 128, 129]. Diekmann and colleagues induced senescence in human dermal fibroblasts and keratinocytes using Mitomycin-C (MMC) treatment and incorporated the cells into OSCs [129]. When compared to mitotic OSCs, the senescent models demonstrated changes similar to aged *in vivo* skin, including a more compact stratum corneum (outer layer of the differentiated epidermis), reduced dermal fibroblast population, decreased collagen type I and III content, decreased elastin expression and looser elastin structures, increases in MMP1, and disordered epidermal differentiation. A similar study involving senescent fibroblasts used healthy fibroblasts that were exposed to H_2_O_2_ to induce senescence and then cultured the senescent fibroblasts in skin equivalents with healthy keratinocytes [128]. Aging phenotypes were characterized by changes in proliferation, differentiation of suprabasal epidermal layers, impairments of skin barrier function, and surface property modification. Further, authors found that fibroblasts exhibited senescence-associated secretory phenotype (SASP) markers including IL-6, GmCSF, and IL-1α. Interestingly, Weinmüllner et al. observed more Ki67 positive epidermal cells when senescent fibroblasts were present. More research is required to understand senescence in the dermis and how it may effect keratinocyte homeostasis [128]. Serial passaging of fibroblasts has also been employed to simulate aging in OSCs, showing that constructs generated with late passage fibroblasts were similar to *in vivo* aged skin [74]. OSCs were generated with 15-20% SA-β-gal positive fibroblasts cells in 2D culture prior to 3D seeding. Authors observed few changes in the epidermal compartment while the dermal component of OSCs presented a thinner dermis and increased MMP1, similar to *in vivo* aged skin [74]. Defects in epidermal-dermal junction in these OSCs were not observed and keratinocytes exhibited a healthy phenotype. Although not shown, authors noted that when greater than 30% SA-β-gal positive fibroblast cells in 2D were used to generate OSCs, the fibroblasts did not produce sufficient extracellular matrix (ECM) and constructs were not viable [74]. As Janson et al. found, generating an OSC using senescent cells is technically challenging since the percentage of senescent cells used to generate an OSC can alter skin structure and long-term culture health [74].

Other studies focused on the aging of the keratinocyte population. In OSCs generated from primary cells isolated from donors, cell donor age is an option for simulating intrinsic aging *in vitro* [71]. OSCs generated with either keratinocytes isolated from aged individuals or serially passaged keratinocyte cells have been used to examine the effects of replicative senescence [130]. Constructs generated with older keratinocytes (61 or 35-year-old donors) exhibited thinner epidermis compared to OSCs generated from 1-year old donor cells. Additionally, there were differences in epidermal organization, where constructs generated with young keratinocytes exhibiting more consistent organization and stratification than OSCs with older cells. This study also investigated the expression of epidermal stem cell markers. They found that when keratinocytes were passaged over six times (modeling *in vitro* cellular senescence), there was a decrease of stemness, indicated by high expression of α6 integrin and low expression of CD71 (a proliferation-associated cell surface marker) [130]. Likewise, in constructs generated with young (infant) keratinocytes, α6 integrin expression was observed in basal cells of epidermis while in constructs generated with adult and elderly cells there was faint and absent α6 integrin expression (respectively). These OSC findings demonstrated in both intrinsic aging (simulated from aged donor cells) and *in vitro* senescence induced by serial passaging results in depletion of epidermal stemness markers [130].

Epidermal changes associated with aging have also been shown in models generated through genetically altering expression of key components, for example p16^Ink4a^ [131]. *In vivo* chronological human aging markers, p16^Ink4a^ and its repressor BM1, are established markers of *in vitro* aging tissue [71, 73, 131, 132]. p16^Ink4a^ is an inhibitor of cyclin-dependent kinases that blocks the progression from G1 phase to S phase of the cell cycle and promotes senescence onset. *In vitro* aged skin models can be generated from young donor keratinocytes cells by p16^Ink4a^ overexpression [131]. Conversely, aging phenotypes observed in old donor keratinocytes can be rescued through silencing p16^Ink4a^. Aged models (both from older donors or p16^Ink4a^ overexpression) resulted in thinner epidermis, loss of stratum corneum (the terminal epidermal layer), and atrophy [131].

OSCs also allow for studies of matrix and cell-matrix interactions in aging skin. Expression patterns of glycosaminoglycans (GAGs) and proteoglycans (PGs) are important in skin tissue mechanical integrity, and aging-related changes contribute to frailty in both intrinsically and extrinsically aged skin [53, 55, 133–137]. Glycation and the presence of advanced glycation end products (AGEs) increase in aging skin, and this has been leveraged in OSCs to create an aged skin model [57, 59]. In this model, collagen was glycated *in vitro* prior to construction of the OSC. This simulated intrinsic aging of the construct, resulting in modified integrin patterns in the suprabasal epidermal layers, activation of the dermal fibroblasts to increase the production of metalloproteinase, type III procollagen, and type IV collagen [57, 79]. Authors found that these morphological and molecular changes in the epidermis and dermis could be partially rescued by antiglycation agents such as aminoguanidine [57]. More investigation is necessary to understand exactly how GAGs and PGs are affected during skin aging. Open questions include how sex specific hormones may affect concentrations [53] and what downstream effects GAGs and PGs have on the expression of cytokines and growth factors [138]. As an accessible platform that can be customized with specific cell lines, biomolecules, and materials, OSCs are uniquely suited to elucidate aging mechanisms including detailed molecular studies regarding GAGs and PGs in skin.

In addition to researching aging biology, OSCs can also be employed as a testing platform for aging therapeutics [135, 138]. C-Xyloside is a xyloside derivative that has been investigated as therapeutic to improve dermal-epidermal junction (DEJ) morphology in aging skin [139, 140]. Sok et al. exposed OSCs to C-Xyloside and investigated the resulting DEJ morphology. C-Xyloside exposure resulted in higher basement membrane protein concentrations, specifically collagen IV, laminin 5, and collagen VII, and organization more similar to the microanatomy of healthy human skin. Further, C-Xyloside increased concentrations of dermal proteins such as pro-collagen I and fibrillin, which are key ECM proteins for the maintenance of skin elasticity. Since defects in the basement membrane, DEJ, or elasticity contribute to skin fragility in aging, this model has potential as a test bed for other aging therapeutics [135].

In the context of skin, tissue engineering has provided accessible and customizable models both for the direct research of aging phenotypes as well as models that can be readily adapted to aging questions. Further, there is demonstrated potential for therapeutic testing. Importantly, the cited models (or variants thereof) rely on commonly available cells, reagents, and techniques adaptable to many lab environments. Increasing use of these models in aging research holds promise to accelerate discovery and therapeutic goals. Despite this promise, there remain challenges to the use of OSCs in aging research, discussed below. Most notably, the power of OSCs comes from their intermediate status between simple *in vitro* models and *in vivo* models; there is an explicit tradeoff between increasing the complexity of the culture system and its cost or ease of use. While OSCs do allow customization by the researcher to focus on factors most important to their question, the tradeoff can be difficult to make for aging research. Some examples of OSC limitations relevant to aging research are provided below.

#### Limitations

The most predominant limitation of using tissue engineered organotypic models is that they typically do not match all cellular populations found *in vivo*. Nerves, sweat glands, stem cell niches, immune cells, subcutaneous adipose, and vasculature are important aspects of aging skin biology that are frequently missing in OSCs. While in many cases there is no strict technical reason for the absence of a specific component, any increase in complexity provides more challenge and cost. For example, inclusion of nerves requires a source of nerve cells, they must be maintained in culture while not losing their phenotype, and simply including cells in the OSC does not capture the complexity of the nervous system. However, progress is being made through iteration, providing researchers with increasingly powerful models that capture more of the relevant physiology. For example, wound healing is slowed in aged skin, and immune cells are vital in both physiological and pathological wounds. While fibrosis has been studied using OSCs, this is typically limited to observing fibroblast and keratinocyte responses; there is a recognized need for OSC models that include immune populations [102]. While not prevalent, some models do incorporate the immune system [104, 116, 141–143], demonstrating the trajectory of the field toward increased capability and flexibility. Similarly, changes in vasculature are prevalent in aged skin, but OSCs often lack vascular cells. While progress has been made in vascularizing OSCs and related models [92, 93, 96, 97, 99, 100, 141, 144–146], there is still a great deal of work to be done in applying this to aging questions.

Further, OSCs tend to be structurally simplified. As mentioned, they typically lack nerves, glands, and other structures typical of skin. Building on the example of vasculature, even with appropriate vascular cells, OSCs often have a random or simplified organization; native cutaneous vasculature is organized into two horizontal plexus planes with connecting vessels between them along the apicobasal axis [147, 148]. In OSCs, this organization could be recapitulated through the inclusion of patterned or semi-patterned vasculature, although this is typically not done [149]. Additionally, decline of collagen density is an important aspect of skin aging, yet many OSCs are fabricated with collagen densities much lower than those found *in vivo* [79, 150]. While not common yet, OSCs can be fabricated from higher collagen densities through techniques such as dense collagen extractions [151], and compression of collagen cultures [152], to more closely represent the *in vivo* dermal matrix.

Another key limitation of current OSCs is loss of systemic factors present *in vivo*. For example, age-associated changes in sex hormone profiles impact skin physiology; e.g., post-menopausal decreases in collagen content, reduced elasticity, and lowered skin moisture in women. While changes in systemic factors can be addressed, they will invariably lack the full complexity of an *in vivo* model. For example, a recent study addressed the impact of exogenous estradiol on elastin synthesis using male and female dermal organotypic cultures [153]. Studies such as this highlight the tradeoffs in organotypic models, as reductionist culture models allow specific questions to be interrogated, they obviously lack the complexity inherent in aging at the organismal scale.

### Intestine/gut

#### Native intestinal aging

**Figure 2:**
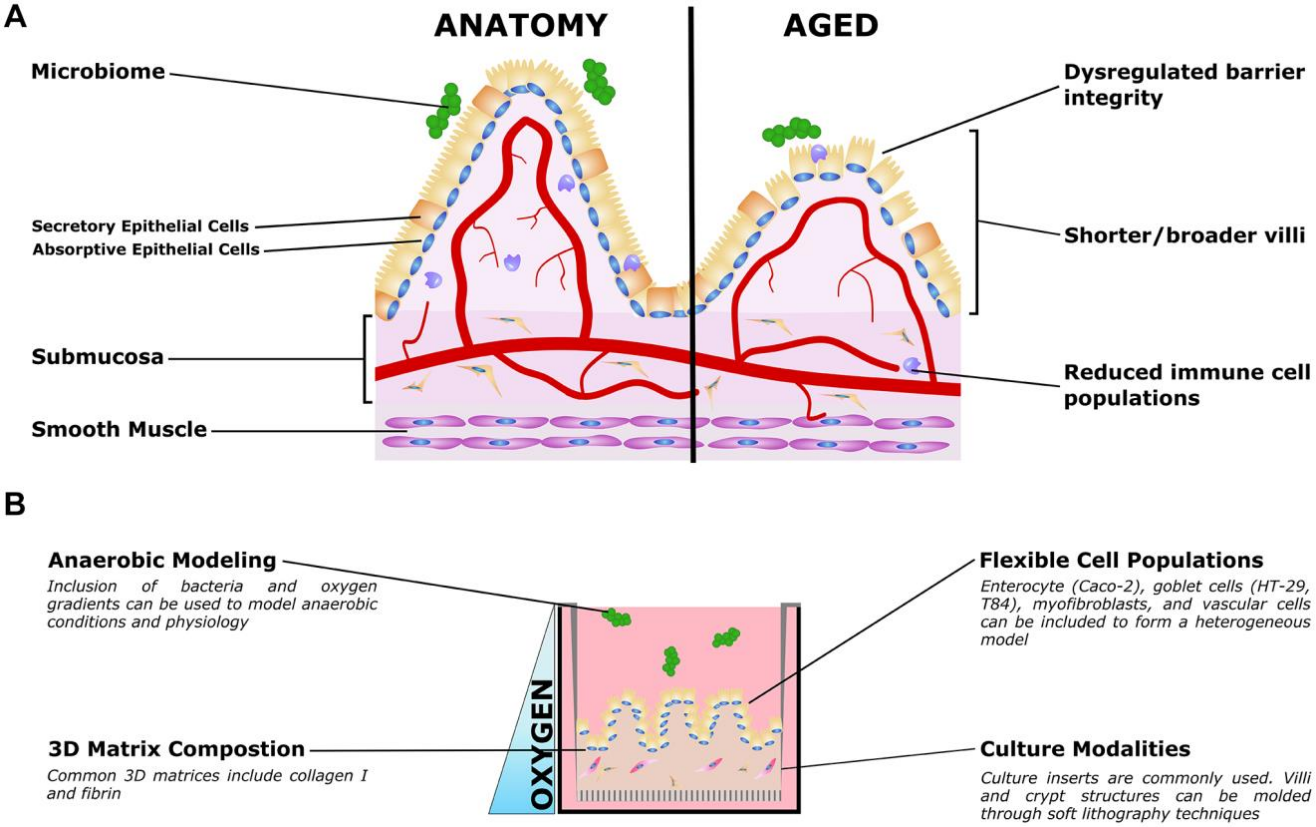
**Organotypic models of gut aging.** (**A**) Simplified gut anatomy and aging, focusing on the most commonly modeled components. A mixed epithelial population, described in the text, forms a simple cuboidal epithelial layer with both secretory and absorptive epithelium. A layer of mucus inside the gut lumen supports the host/microbiome interaction. The stroma underneath the epithelium, the submucosa, is host to nerves (not shown) blood vessels, fibroblasts, and immune cells important for gut function. Smooth muscle is required for gut peristalsis. In aging, the macrostructure of villi degrades, with villi becoming shorter and broader. Immune cell populations are disrupted, and reduced epithelial barrier integrity can lead to increased microbial infiltration into the submucosa and vasculature. (**B**) Organotypic models of the gut typically only model a small subset of these features, and are typically adapted to aspects that are relevant to specific questions. For example, epithelial and immune populations may be co-cultured to study intercellular interactions in a simple format. To study the influence of villous structures, soft lithography can be used to recreate the villi/crypt geometry. Microbiome co-cultures can be included, and microfluidic organ-on-a-chip models have been used to mimic the oxygen gradient from the vascularized submucosa to the anaerobic lumen.

In this section we focus on the gastrointestinal system and review relevant three-dimensional organotypic culture models. The small intestine is the primary organ for nutrient absorption from food, while the colon (or large intestine) is the primary organ for reabsorption of water [154]. Here, we focus on the small intestine, due to the larger number of *in vitro* three-dimensional models, but large intestine models are briefly discussed as well. The small intestine has a complex tissue structure involving crypts (valley points) and villi (mountain points); with the crypts providing a stem cell niche (Figure 2A). Stem cells located within crypts asymmetrically divide and the resultant epithelial cells migrate up toward villi and eventually slough off into the gut lumen. Multiple distinct epithelial populations arise from these stem cells, including microfold cells, enteroendocrine cells, enterocytes, goblet cells, Paneth cells, and tuft cells; this process of continual epithelial renewal and differentiation is integral to a healthy gut barrier. On the epithelial surface there is a brush boarder and single or bi-layered mucus layer depending on location within the gut [155]. Interacting with this surface is the microbiome which is made up of commensal bacteria and pathobionts (resident microbes with pathogenic potential) that constantly interact with the mucin layer of the gut [155]. Diversity of the gut microbiome has been established as an important factor in gut health and host health [156–165]. The diversity of the microbiota presents in different regions of the gastrointestinal tract depend on many factors including pH, host health, mucin composition, bacterial cooperation, nutrient availability, location within the gut, and age of the host [157]. Further, within the subepithelial and stromal tissue there are additional cells, including fibroblasts, smooth muscle cells, microvascular cells, and both circulating and resident immune cells (e.g., monocyte derived macrophages, neutrophils, dendritic cells, T cells). The immune cells are known to interact with and traverse the epithelial surface [166–168]. Given the complexity of the intestinal tissue and the number of host and bacterial cell types, it is unsurprising that many of the cellular interactions are poorly understood, especially in aging tissue where both the host tissue and microbiome can change [169].

Aging in the gut presents as reductions to nutrient ingestion, the tolerance of resident microbiota, and the response to infection (key aging phenotypes are summarized in Table 2). Often these co-present with dehydration and malnutrition [166]. Generally, there is a lower intake of macronutrients and micronutrients in aged individuals, although this lower intake could be attributed to lower physical activity, problems with teeth, impaired sense of taste and smell, psychological factors, income levels, and drug side effects [170–172]. Together, lessened nutrient intake, dehydration, and malnutrition contribute to overall healthy decline and morbidity in aged individuals [172]. Additionally, there is evidence showing that absorption of glucose and vitamins increases with age while some nutrients such as cholesterol and fatty acid decrease or slow; changes in absorption have been well reviewed in animals [170, 172] but continues to require more investigation in the human gut [172, 173]. It has been suggested that changes in nutrient absorption could also be tied to the changes in morphology found in aged animals and in humans [174].

**Table 2:** Prominent phenotypes of aging intestine.

**Prominent aging phenotypes**	**References**
Increased microbial infiltration into submucosa and vasculature	[180–182]
Reductions to nutrient ingestion, tolerance of resident microbiota, and the response to infection.	[166]
Villi morphology changes, decreased cells per villus, decreased mucosal surface area, decreased crypt numbers	[174, 177, 178, 183–185]
Increased cell apoptosis, reduced cell proliferation and survival, decreased regenerative potential of stem cells	[166, 177, 184–187]
Disruption of Wnt Signaling	[177, 188–190]

Morphologically, as the small intestine ages, numerous structural changes have been observed in several models. These structural changes are coupled to cellular changes, for example, the dynamics of cell life cycle from the crypt to extrusion at the villi [170, 175–177]. In one year old rabbits compared to young rabbits, there are morphological changes in the jejunum and ileum; villi shorten, number of cells/villus drops, and mucosal surface area declines in the jejunum while villus cell size remained constant in both areas [178]. Changes in villous height are associated with mucosal surface area at all ages [178] and these declines in surface area have been related to differences in nutrient absorption of aged individuals [174]. In healthy mice it takes around 4-5 days for a stem cell derived progenitor to move from the crypt, differentiating along the way, to the tip of the villus, where it ultimately undergoes apoptosis and extrusion. Morphological changes such as villi length increase and crypt number decrease lead to larger crypts with more cells and are coupled with less travel of progenitor cells to the tip of the villus as well as increased apoptotic events, decreased cell proliferation, and lower cell survival in aged mice [177]. Aging and how it effects wound healing in the small intestine has also been investigated in mouse models. Martin and colleagues studied the regenerative capacity of small intestinal epithelium after injury in young and old mice using full or partial body irradiation [179]. Authors found that after injury induced by full body irradiation, crypts of old mice were smaller than controls while young mice had larger crypts. After partial body irradiation, the crypts of young animals were found to be smaller, while the number of surviving crypts in old mice was lower than in young mice.

In rats, morphological changes such as increased numbers of crypts and villi are observed with aging, although size and cell production rate changes were not observed [183]. Atrophy of intestinal mucosa also occurs in aged rats and this contributes to decreased number of enterocytes [184, 185]. These changes can be localized to specific tissues; for example, mucosal atrophy in rats has been found in proximal regions of the small intestine, but not in the distal small intestine; similarly, the decline in villi height has been found in the ileum but not the duodenum [184]. Changes in morphology are thought to be closely tied to transport function across the gut barrier and may be tied to malabsorption of nutrients, but more evidence is needed to support this [169, 170, 174, 178]. Further, the association between aging and morphological changes is poorly understood in human intestine. Currently, there are few studies that have examined human intestinal morphology; Webster and colleagues found that elderly people have shorter villi and possibly broader villi when comparing shape and dimensions of proximal jejunal villi in young versus aged humans [174]. The villous changes in humans were not definitively linked to changes in intestinal function, but changes in surface area are thought to contribute to the nutrient absorption decline that aged individuals often experience [174].

Changes in enzyme distribution and brush border membrane makeup have been observed in mice [170], rats [185], and rabbits [178], but the conclusions differ by species and it is unclear whether these changes are associated with aging [170]. Briefly, in adult and aged mice there are similar activities and distribution of enzymes in the brush boarder membrane [170]; while in aged rats lower alkaline phosphatase activities have been found; conversely, higher sucrase/alkaline phosphatase in the brush boarder membrane have been found in adult vs. young rabbits. Differences in mucus structure and chemical composition have been tied to age changes [166, 170, 191]; specifically glycoproteins in the mucus change with age in rats [170, 191]. There is some evidence suggesting that the process of bacterial adhesion to mucus also changes with age, shown with bifidobacterial strains [166, 192–194]. However, gastric and duodenal mucus thickness does not change with age in healthy individuals [166, 195]; mechanical properties of mucus have been found to remain stable as well [166].

On a cellular level, differences have been observed with aging. Most prominently, stem cell changes have been observed in aged animal studies and in organoid cultures [177, 188]. In small intestinal tissue from mice, the intestinal stem cell markers *Lgr5* and *Olfm4* were examined but found to be similar in young and old samples, while the quiescent intestinal stem cell markers *Lrig1* and *Tert* were reduced [177]. However, when examining numbers of stem cells in young versus old cultures, no difference was found [177]. Wnt signaling, an important aspect of self-renewal and proliferation in intestinal stem cells, is altered in aging gut [188–190]. Elevated Wnt activation can lead to intestinal tumorigenesis [196] and malformed crypts (less lobes and buds per crypt) in small intestine mouse organoid cultures [189]. However, there is conflicting literature on how elevated or lowered Wnt signaling effects stem cells in aged mice. Nalapareddy and colleagues found that during aging, intestinal stem cells, Paneth cells, and mesenchyme secrete less Wnt ligands which leads to overall reduced Wnt signaling and lower regenerative potential of stem cells [177]. Using organoid models derived from duodenal (proximal) crypts in mice, the decreased stem cell function can be rescued by endogenous Wnt *in vitro* [177]. There is evidence that the stem cells may lose fitness in maintaining differentiated cell populations; specifically Paneth cells, responsible for generating anti-microbial peptides [166]. The amount of Paneth cells and their secretory functions have been found to decline with age [166, 187], and this may be due to the age-related stem cell decline and reduced ability to generate Paneth cells [166, 179, 197].

The mucus is the site of antibody production (specifically, secretory immunoglobulin A; IgA) and is the first defense against harmful microorganisms [166]. Goblet cells, the primary contributor to the mucus layer, have a stable population in aging mice [166, 198]. As previously reviewed, the literature remains unclear on the effect of aging on IgA response, migration, and production [166]. Aging has been found to decrease secretory IgA amounts in animals (mice, rat, non-human primates) when exposed to cholera toxin [166, 199–202] and increase somatic hypermutation in mice [166, 203]. In contrast, other studies have shown no changes in serum or intestinal amounts of IgA in aged rats and mice; some results suggest that the lower levels of IgA are due to an overall homing decline rather than changes in amounts of IgA [166, 201, 204–207]. Dendritic cells present antigens to B and T cells in the intestinal immune system, and evidence points to decreasing cell numbers and function in aged mice [186]. Further, this plays a role in decline of regulatory immune functioning [166, 208, 209] and may play a role in low grade inflammation observed in the aging gut [166, 169, 210, 211].

The microbiome plays an important role in digestion, absorption, and nutrient processing [212], but it remains incompletely understood how the intestinal barrier and immune system interact with microbiota and how this system is affected by aging. In the study of microbiota, it remains unclear how gut diversity affects the aging process and how gut diversity changes with age. There is not enough evidence or investigation on age-related associations and gut health to determine causes/effects of gut on old age [164, 165], although there are many health practices that correlate with perturbations of the gut microbiome including drug/antibiotic usage and diet [164, 213]. There is evidence that the gut microbiome is affected by sex differences [212, 214–217], and this may be implicated in sex differences in aging-associated disease. Sex differences in the microbiome affect gut health but also risk of disease development including atherosclerosis, diabetes, hypertension, dyslipidemia, and obesity [212]. In general, aging and its relation to sex and hormonal differences requires more investigation, but there are indications that changes in the aging gut are sex-linked due to hormonal differences during early life, adulthood, and aging [214, 215]. In aging males, testosterone levels drop slightly from levels during adulthood while in aging females, there is a dramatic drop in estrogen from the oscillation range of adulthood [215]. The general effects of hormonal supply decline to the gut microbiome are unknown, but are likely sex-specific [215] and may be associated with the immune component of the gut [216].

#### Tissue engineered gut models

There are a few limitations to traditional intestinal models that can be addressed with 3D organotypic gut models (Figure 2B). 2D cultures on culture inserts are often used to model gut, but these cultures are unstable after 4 weeks due to cellular overgrowth and formation of multicellular layers [155]. To study enteric bacterial pathogens, researchers have often used human tissue explants; animal models [218]; and 2D cultures with cell lines such as T84 and HT-29 which mimic goblet cells, and Caco-2 which serve as enterocytes [219]. Although helpful in understanding microbiome-host responses, these models are typically inconsistent with the human anatomy and physiology in the gut [218, 220]. Similarly, mouse transgenic models are often used to study inflammatory gut diseases but mice do not develop some prevalent human diseases, such as ulcerative colitis or Barrett’s esophagus [221]. To address gaps in more traditional models, several 3D models have been established based on organoid, explant cultures, micro-fluidic chips, and organotypic gut models (OGMs) generated through self-assembly and partial villous molding. Intestinal tissue derived organoids are a popular model that has been used to study aging; these are called enteroids for small intestine, or colonoids for large intestine models. Enteroids consist of only epithelial cells and model crypt like populations or are often differentiated to model surface/villous epithelium [218]; these have been studied using monolayers on tissue culture inserts and embedded in extracellular matrix [218, 221]. Human induced pluripotent stem cell (iPSC) derived intestinal organoids, contain both epithelial and mesenchymal lineages and model both crypt and surface villus [218]. Models of differentiated intestinal organoids, although limit appropriate human scale, can include even the rare cells of intestine models including enteroendocrine, tuft, M cells, and Paneth cells [222].

3D cultures have been generated with both primary human cells and commercially available lines. OGMs have been generated with adult human intestinal stem cells [222], iPSC [222], Caco-2 [155, 222, 223], T84 [222], HT-29 [155, 222, 223], and myofibroblasts [155]. OGMs are only recently developed, but they have advantages over 2D models, micro-fluidic chips, explant cultures, and organoid structures because of their ability to mimic appropriate tissue length scales for oxygen diffusion and customizable cell and material properties [218]. Additionally, human based models that include human cells and relevant 3D microenvironments can be used to study diseases such as gastroesophageal reflux disease, Barrett’s esophagus, IBD, and ulcerative colitis; for therapeutic screening; and other aging associated research [221].

Incorporation of 3D villi in OGMs have been demonstrated to model the human system more closely [220] and help to understand the changes in crypt/villi that have been observed in aged animals [177, 178, 183, 184]. Several groups have generated 3D gut models with villous platforms though pre-culture molding of hydrogels and custom plate inserts [219, 220, 224]. These systems have been found to mimic mammalian intestines more closely than 2D cultures facilitating cell differentiation, absorption/metabolism, and have been used to evaluate drug permeability [220]. Yi and colleagues compared absorption and metabolism of enterocyte (Caco-2) 2D monolayer cultures and 3D villous collagen scaffolds covered with enterocytes. They found that in the 3D cultures, cell growth was higher (likely due to more surface area), there were more *in vivo* phenotypes such as lower expression of P-gp (efflux transporter protein, p-glycoprotein) which is overexpressed in 2D monolayers, and increased alkaline phosphatase expression (a metabolic enzyme and intestinal epithelial differentiation marker) [219]. To generate 3D collagen villi structures, multiple groups have used relatively stiff collagen and an alginate reverse molding method to create villous structures from collagen hydrogel [219, 220]. Yu and colleagues promoted a basement membrane like surface by coating the collagen with laminin. Villous structures were fabricated to match the density and depth of human villi and models were cultured for 14 days; a 21-day duration led to breakdown of villi [220]. Similar pre-culture molding of villous structures has been used in microfluidic-chips [225–227]; and as reviewed by others [225]. These models capture appropriate microanatomy of the intestinal surface and have the potential to elucidate the respective roles of structural and cellular changes in aging.

Organoid models have been used to study several diseases [189, 190, 222, 228, 229]; illustrating how 3D cultures provide a physiologically relevant model without the complexity of fully *in vivo* studies. Woo and colleagues demonstrate how a 3D model (specifically an intestinal organoid spheroid model) can be used to study the human disease dyskeratosis congenita. Dyskeratosis congenita causes intestinal defects (including stem cell failure) and is characterized by decreases in telomerase, telomere length, telomere capping, and Wnt activity [190]; it is particularly relevant to aging since some of these disease characteristics are similar to what happens in aged intestinal cells [188]. In organoids generated with the dyskeratosis congenita model cell line, there was incomplete and thin epithelia, overgrowth of mesenchymal cells, and inferior E-cadherin and beta-catenin expression; the organoids did not have proper budding crypts or cavitation [190]. Through CRISPR/CAS9-mediated repair and administration of Wnt agonists the authors were able to rescue the disease phenotype and demonstrate normal organoid formation *in vitro.* In other disease specific models, organoids made with cells derived from inflammatory bowel disease patients maintain characteristics of disease *in vitro* such as gene expression profiles that regulate absorption and secretion [222, 228]. Disease focused organoid studies [190] and other organoid models generated with aged mice cells [189] demonstrate the potential of more physiologically relevant *in vitro* models to address aging questions. By building off of these methods and incorporating human cell types, anatomies, and physiology it is possible to develop a human derived organotypic gut model [155] and avoid costly procedures involved in animal colonies [213].

#### Tissue engineered gut models to study aging

A recent study by Arnold and colleagues demonstrates the physiological relevance of 3D *in vitro* models for aging [230]. *In vivo*, older animals have higher ratios of non-saccharolytic v. saccharolytic bacteria and lower amounts of β-galactosidase when compared to younger animals. Pre-biotic galacto-oligosaccharides (GOS) have previously been found to have a positive impact on intestinal health and can be administered through diet. To study the effects of dietary GOS on aging in the gut, using young and old mice models of *Clostridiodes difficile* were used. In the aged mouse models, dietary GOS promoted changes in microbiome composition and transcriptomic analysis also revealed differences in gene expression. Aged mice that were fed a GOS diet had decreased intestinal permeability and increased mucus abundance and thickness when compared to aged mice not fed the GOS diet. These changes in permeability supported previous findings attributing the leaky gut to increased non-saccharolytic bacteria and lower amounts of key enzymes. Further, these results were additional tested in colonic organoids injected with stool samples from young and old mice. Using the colonic organoids generated from one young mouse and stool sample injection from experimental mouse models, authors showed that they were able to reproduce differences of age, minor differences of the GOS diet, and bifidogenic responses observed in the *in vivo* mouse models [230]. As the authors already showed a reproduction of aged phenotypes in organoid models, reproducing these characteristics in scalable and humanized organotypic models may be beneficial in research questions of how diet and microbiome affect aged humans.

The ability to culture anaerobic bacteria is an important step in modeling the microbiome of the gut in healthy tissue and to improving the understanding of how aging changes the host-microbiome interaction [158, 163, 164, 231–233]. Most *in vitro* models, including OGMs, only study a few relevant features of the complex physiology at a time; models that include microbiota are no exception. One study showed their ability to culture 5 different microbe types *in vitro* on a custom scaffold and evaluated for proliferation and biofilm formation [234]. It is important to recognize, that although this is a human microbiota gut model, it does not incorporate human gut cells or microanatomy. Combining microbiota and human 3D OGMs is an important step in modeling the human gut; some work on the combinations of microbiota and human gut cells has been carried out in microfluidic chips [225], but these tend to lack relevant villous anatomy and appropriate oxygen diffusion scales. These factors have been partially addressed in an innovative upright cylindrical culture system [155]. Authors generated the vertical lumen with an un-patterned surface and a threaded surface to mimic crypt and villi of the intestine. Their model includes epithelial cells (Caco-2 and mucus producing HT-29 cells) and myofibroblasts seeded on and into silk-based scaffolds, respectively. With this design, they achieved proximal-to-distal oxygen gradients and reached anaerobic conditions in patterned lumens. As a proof of concept, they cultured anaerobic bacteria using this model. Importantly, the patterned lumen model was stable for long-term culture (at least 8 weeks); they further showed continuous mucus production and accumulation (~10 μm average thickness of the mucus layer). Although this model does not incorporate aging phenotypes, aged cells, or differences due to aging in the microbiome, it highlights the recent progress in developing organotypic constructs that could be adapted to aging studies.

*In vitro* organoids are common in the gut/microbiome field of study [188, 218, 222, 235, 236] and have been used to assess intestinal stem cell function during chronological aging [177, 188–190, 237, 238]. Although there is conflicting literature on Wnt signaling in the intestine and how it effects intestinal stem cells, several recent studies have used organoid models to investigate aging and how it changes crypt/villi formation and stem cell function in the gut. Each study also presented a rescue method to restore normal Wnt signaling and gut formations [177, 189]. Cui et al. cultured organoids from aged mice and showed reduced differentiation and increased expression of Wnt target genes (*Axin2* and *Ascl2*). The organoids generated from aged mice presented rounded cysts without typical differentiated cell types, in contrast to organoids generated from young mice, which demonstrated differentiation and formation of villus structures. These phenotypes matched organoid cultures of cells that exhibit overactivation of Wnt signaling (through seeding with adenomatous polyposis coli deficient cells). The decreased differentiation of intestinal stem cells and impaired structure could be rescued by reducing exposure to the Wnt agonist R-spondin-1 and thus reducing Wnt activity. Rescued organoids matched those generated with cells isolated from young mice. Nalpareddy and colleagues generated organoids from duodenal proximal crypts of aged and young mice as well as humans [177]. In humans, organoids were generated from people 12–16 and 62–77 years old. The authors found decreased formation of organoids in the aged group, which was improved by adding Wnt 3a (a Wnt pathway agonist). This data supported their findings in mice organoids where aged mice organoids had lower organoid formation rates after 3 passages and decreased stem cell function (determined by lower lobes and buds per crypt). Adding Wnt 3a increased organoid formation and expression of Wnt target genes (*Axin1* and *Ascl2*) in the aged cultures [177]. While interpreting the apparently contradictory results of these studies is difficult, they do highlight the use of organotypic models in performing detailed signaling studies that would be challenging and expensive in animal models.

*In vitro* intestinal models have a particularly relevant potential impact on personalized medicine due to the person-to-person variability in gut health. Aside from genetics, variation in local community and world regions as well as day-to-day activities result in microbiome and inflammatory differences that are not yet understood [239]. Personalized medicine and patient derived organotypic models may help to address these parameters. One organotypic microfluidic chip model named iHuMiX has paved the way for personalized gut models [240]. The iHuMiX platform utilizes compartments including microbial, epithelial, and flow chambers and allows for study of specific bacteria on host specific physiology. While microfluidic systems often present technical barriers for non-specialist labs, these results highlight the customizability of organotypic models, including adaption to personalized medicine. As with OSCs described in the prior section, the tradeoff between complexity and capability for organotypic gut models results in several limitations.

#### Limitations

As with OSCs and other organotypic models, the most prominent limitation is the lack of cell populations and structural features of the *in vivo* gut. While a great deal of the work described above has extensively modeled epithelial cells and their stem cell niches, the gut is much more complex; immune cells, vasculature, smooth muscle, and neuronal populations all contribute to the gut, and its physiology when aged. Further, the organization of the gut, most notably the crypts and villi, is well understood to influence function and disease; these features are only incompletely reflected in organotypic models [219, 220, 241]. More unique to the gut is the anaerobic microbiome, which is critical to understanding gut and organismal health [158, 163, 164, 231–233]. While there has been demonstrated inclusion of anaerobic microbiome in a gut model [225–227], the complexity of the system makes it challenging to broadly replicate in other labs. Indeed, the general challenges of creating and maintaining hypoxic and anoxic cultures significantly limits the ability of organotypic models to correctly match the lumen environment. Further, there is significant evidence that the microbiome is not restricted to the gut lumen, and translocation of commensal bacteria to surrounding tissues, including lymph nodes, is a driver of disease [242, 243]. While organotypic gut models may be suited to address some questions of bacterial translocation, none have reached the scale or complexity required to include lymphatics. While this is a single example, it does highlight the more general limitations on most organotypic models.

As with other organotypic models, sex differences are understudied. This is despite clear sex differences in aging associated gastrointestinal diseases [244, 245] and cancers [246, 247]. While sex differences local to the cell populations used could, and should, be studied using organotypic models, systemic factors including hormones remain a challenge. As a pertinent example in the gut, sex hormone levels are known to regulate the mucosal surface and barrier integrity [248]. While organotypic models to lend themselves to studying the impact of specific hormone levels, they clearly lack the complexity of overall systemic changes that come with aging and sex differences.

### Skeletal muscle

#### Native skeletal muscle aging

Skeletal muscle is an abundant tissue, making up ~30–40% of body mass [249]. Healthy muscle regulates major physiological processes such as locomotion [250, 251], venous return [252–254] and metabolism [255–258]. From the 3^rd^ to 8^th^ decade of life fat-free mass declines by ~15%, even for healthy individuals, contributing to loss of independence and higher risk of injury and mortality. The age-associated loss of muscle mass, known as sarcopenia, is a major hallmark of human aging [259–261] with a complex etiology, resulting in muscular, vascular, and metabolic impairment [262–264]. Chronic inflammation [265–268], nutrient deficiencies [269–271], and decreased physical activity [272–274] are all contributing factors of sarcopenia, however, much remains unknown at the molecular, cellular, and tissue levels. Improved models of sarcopenia and other aging phenotypes are imperative for improving clinical outcomes and prophylaxis for the expanding geriatric populations.

**Figure 3:**
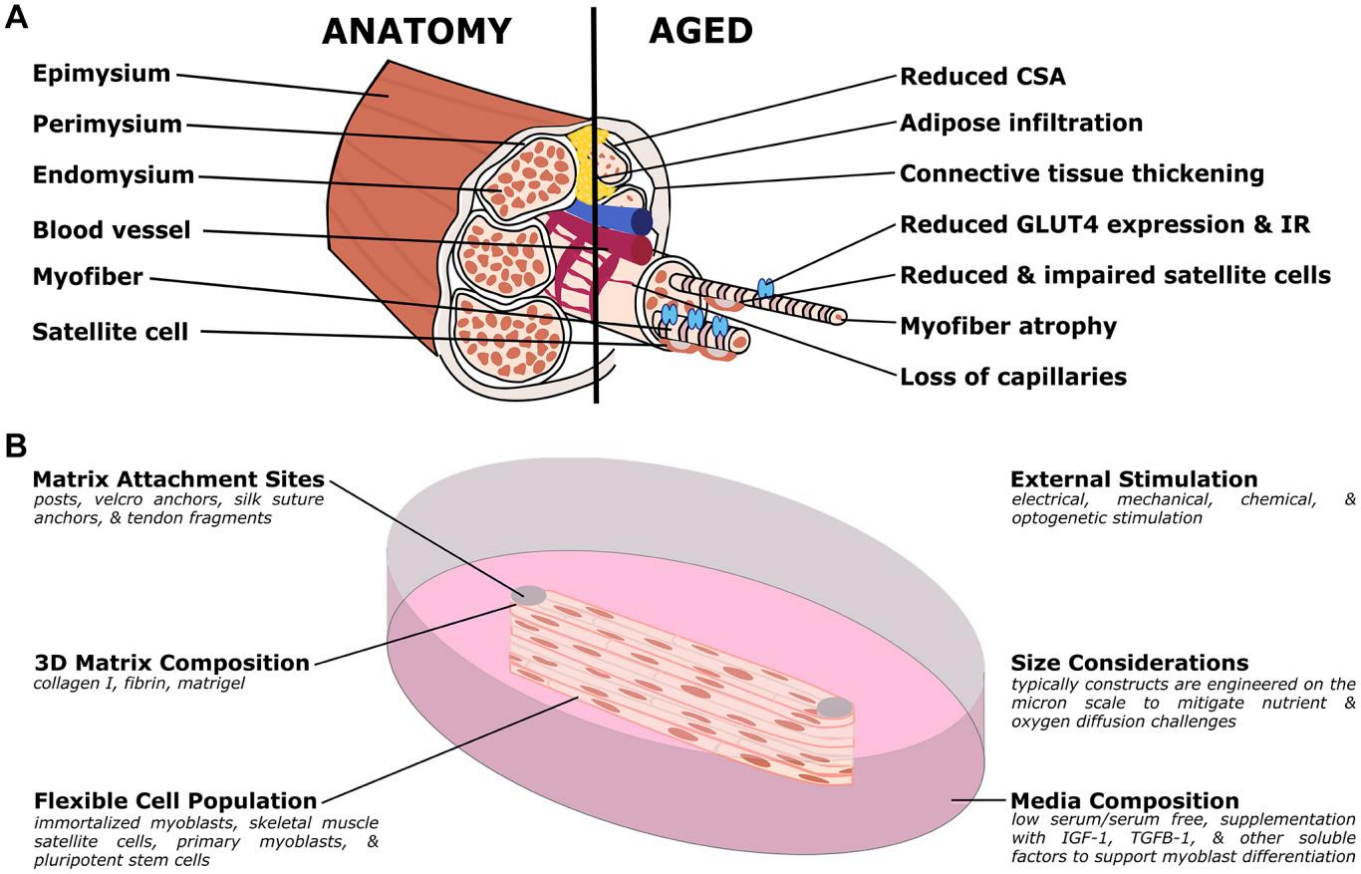
**Organotypic models of skeletal muscle aging.** (**A**) Simplified muscle anatomy and aging, focusing on the most commonly modeled components. The primary unit of muscle is the myofiber, a multinucleated cell responsible for contraction. Specialized matrix (endomysium, perimysium, and epimysium) support and organize the tissue. Satellite cells are an important stem cell population for the muscle, and the muscle is supported by a host of other cell types including nerves, fibroblasts, adipose, and vascular cells. In aged muscle, cross-sectional area (CSA) is reduced, in part due to myofiber atrophy, and decreasing capillary and satellite cell density. Conversely, there is increased infiltration of adipose and thickening of the connective tissues. At the molecular level, there is decreased expression of GLUT4, an important glucose transporter, and insulin resistance (IR) frequently develops. (**B**) Organotypic models of muscle have several unique challenges but have distinct advantages over other traditional models. Muscle cultures are contractile, and require anchoring to prevent collapse. Typical approaches include posts (although other methods are used) to provide points of resistance for the muscle to pull against. In order to study active contraction, researchers have used various stimulation methods, including electrical and optogenetic methods. Due to the high metabolic demand, the cultures are typically quite small, to allow nutrients and waste to diffuse more readily. As with other organotypic models, the matrix, cell population, and media can be customized for the research question.

In a healthy individual, skeletal muscle is composed of densely packed and aligned cylindrical myofibers individually sheathed in a specialized matrix called endomysium [275] (Figure 3A). Bundles of myofibers are encapsulated in a connective tissue layer known as the perimysium, while the whole muscle is surrounded in a thicker connective tissue layer called the epimysium. Myofibers are organized into fiber types (fast twitch and slow twitch) based on their metabolic, contractile, and morphological properties. Due to the unique signature of each fiber type, maintaining homeostatic fiber compositions is vital to muscle function [276]. Multiple muscle fibers and the corresponding motor neuron form a motor unit, with the overall force of muscle contraction controlled by activating more motor units. A dense vascular network that delivers nutrients and removes waste supports the high metabolic demands of muscle tissue.

Structural and cellular changes are prominent in aged muscle (summarized in Table 3). Structural changes include reduced muscle cross sectional area [277–280], thickening of the epimysium and endomysium connective tissue layers [281–284], increases in tissue fibrosis [285, 286], and decreased capillarization [278, 287, 288]. Further, reduction and atrophy of specific fiber types (particularly fast twitch/Type II fibers) has been observed, leading to altered fiber composition and increased percentages of slow twitch (Type I) fibers [289–292]. More specifically, Type II (fast) fiber atrophy is associated with reduced muscle mass and strength [289, 293]. Cellular changes include increased adipose infiltration into the muscle [294–296], and loss of motor units [297–299]; all result in decreased skeletal muscle force generation. Further, age associated changes in skeletal muscle satellite cell populations include a reduced progenitor pool [300–302], limited myogenic colony formation [303], loss of amplification and myofiber differentiation potential [285, 304–308], and an increased susceptibility to senescence and apoptosis [301]. Further, aged satellite cells have been shown to favor fibroblastic and adipogenic differentiation programs [285, 309–311], potentially explaining the observed increase in fibro-adipogenic progenitors in aged skeletal muscle [312–314]. Of course, aging muscle includes non-muscle cells, other skeletal muscle aging phenotypes include increased M2 macrophage presence [315–317] and endothelial apoptosis [318]. Together these cellular and microstructural changes contribute to loss of muscular and systemic function in the elderly population, motivating research into the molecular mechanisms underpinning these changes.

**Table 3:** Prominent phenotypes of aging skeletal muscle.

**Prominent Aging Phenotypes**	**References**
Myofiber atrophy, reduced cross-sectional area, reduced mass, loss of motor units, and decreased strength	[277–280, 289, 293, 297–299]
Change in the ratio of fiber types (increased percentages of slow twitch/Type I fibers)	[289–292]
Decreased vascularization and increased endothelial cell apoptosis	[278, 287, 288, 318]
Increased fibrosis and thickening of connective tissue layers	[281–286]
Increased adipose infiltration and differentiation	[309–314]
Decreased progenitor pool and loss of regenerative capacity	[285, 300–302, 304–308]
Increased insulin resistance and metabolic dysfunction	[319–325]

The above structural and cellular changes are coupled with molecular changes in the aged tissue. A loss of overall regenerative potential is likely largely influenced by a reduced satellite cell population and differentiation potential [306, 308]. Satellite cell activation is regulated by myogenic regulatory factors (MRFs). Primary examples of MRFs include: myogenin, myogenic determination factor (MyoD), myogenic factor 5 (Myf-5), and myogenic regulatory factor 4 (MRF4) [326]. In rats, MyoD and myogenin have been found to increase with age, indicating a potential compensatory role to attenuate loss of satellite cell activation [327]. Yet, human studies have observed a decrease in myogenin, Myf-5, and MyoD [328, 329]. Differential responses between organisms such as this emphasize the need for robust models of human muscle tissue. Myostatin, a member of the TGF-β superfamily, inhibits satellite cell proliferation (via upregulation of p21) and activation (via reduced MRF expression). Further, the elevation of myostatin contributes to muscle atrophy through glucocorticoid signaling [330–332]. Upregulation of myostatin is seen in aged individuals and is thought to contribute to age-associated loss of muscle mass [333–335]. Further, mitochondrial dysfunction and increased oxidative stress are hallmarks of aged muscle [336–339]. Mitochondria manage the cell’s energy supply, ROS generation, and apoptosis. Changes in mitochondrial bioenergetics lead to ROS accumulation, impaired quality control mechanisms, and apoptotic cell death [340–342]. ROS accumulation in aged muscle mitochondria contributes to protein and DNA damage [343–346]. This subsequent loss of mitochondria quality control mechanisms establishes a feedforward cycle of mitochondrial damage and muscle degeneration [347].

Of course, muscle is not separate from the systemic context, both being influenced by and influencing changes in the entire aged organism. Systemic changes contributing to skeletal muscle aging include altered cytokine and hormone signaling. Insulin-like growth factor (IGF) is both a circulating hormone and localized growth factor. IGF is predominantly produced by the liver and delivered systemically, although other tissues produce specific IGF splice variants; mechano growth factor (MGF) and IGF-1Ea are produced by skeletal muscle [348–350]. In skeletal muscle, IGF regulates muscle hypertrophy and growth, and concentrations are known to decline in elderly populations [327, 351, 352]. IGF and MGF are responsible for activating anabolic and anti-catabolic pathways via PI3K/Akt, ERK/MAPK, and PKC signaling, leading to increased protein synthesis and anabolic activity [351–353]. Examples of aging-associated dysregulation of IGF signaling includes evidence that mechanical loading of skeletal muscle results in MGF stimulation in young individuals, but not the elderly [354]. Inflammatory cytokines are also implicated in muscle aging. Elevated TNFα concentrations are found in aged muscle and cause increased apoptosis [355]. IL-6 is a pleotropic cytokine known to influence skeletal muscle function in a number of ways [356]. Elevated levels of IL-6 are strongly associated with diseased muscle, proinflammatory signaling, and a catabolic shift. In rats, with positive stress stimuli such as physical activity, IL-6 levels increase and may have anti-inflammatory effects [357]. In the context of aging there is evidence that in aged human muscle, chronically IL-6 elevated can initiate muscle wasting [358]. In contrast, local IL-6 expression appears in both young and aged individuals after exercise with beneficial effects, indicating a complex role for IL-6 in muscle homeostasis [359, 360].

Hormonally, testosterone and its precursor, dehydroepiandrosterone (DHEA), are key regulators of muscle mass. Androgens (including testosterone and DHEA) are important for maintaining muscle mass through hypertrophy via increases in myonuclear number and fiber cross-sectional area [361–363]. The mechanisms driving androgen mediated muscle growth are poorly understood, but there is evidence of impact on satellite cell commitment level and trophic signaling, discussed in more detail in other reviews [361, 362]. Relevant to the present work, androgen levels decrease in the elderly and contribute to reduced muscle mass [362, 364–367]. Thyroid hormones (TH), T_3_ and T_4_, are important regulators of metabolism, contractile function, and muscle differentiation [368, 369]. Expression of TH decreases with age [370], and this may be involved in the development of sarcopenia [371, 372].

Skeletal muscle also regulates systemic AAD. Skeletal muscle insulin resistance is a primary characteristic of Type II Diabetes (T2D) that presents years before the disease’s onset [323–325]. Yet, the mechanism connecting the pathogenesis of T2D and skeletal muscle insulin resistance is incompletely understood. Increases in mitochondrial dysregulation, oxidative stress, and inflammation are all known to contribute to diminished insulin sensitivity in skeletal muscle. Indeed, it has been demonstrated that elderly individuals have impaired glucose metabolism, and decreased expression of the insulin-mediated glucose transporter, GLUT4 [320–322]. Additionally, aged skeletal muscle exhibits reduced rates of mitochondrial oxidative phosphorylation and an inability to switch from lipid to glucose oxidation when stimulated with insulin [319]. Reduced insulin sensitivity of aged muscle contributes to the development of diabetes and other metabolic disorders. Importantly, the above molecular changes are not broadly conserved across species and gender, emphasizing the need to ensure research models match the morphological, functional, and biochemical characteristics observed *in vivo*. Overall, understanding human skeletal muscle aging remains a challenge, especially considering the diverse and interacting factors at the molecular, cellular, and tissue scales. Developing models that mimic the native tissue, while remaining accessible to experimental techniques, are needed to further push the field forward.

#### Tissue engineered muscle models

Tissue engineered skeletal muscle models, pioneered by Vandenburgh and colleagues [373], have been in use for over two decades. The earliest engineered constructs, termed bioartificial muscle (BAM), consist of skeletal myoblasts encapsulated in an ECM. The ECM is molded around artificial “tendons”, or posts, responsible for maintaining passive tension within the tissue (Figure 3B). As the myoblasts differentiate into highly contractile myotubes the cells align along the axis of tension and lift off the culture substrate. Myoblasts from a range of developmental stages are commonly sourced from muscle biopsies of organisms such as avian [374], mouse [375, 376], rat [377–379], and human [380–383]. Due to limited availability of primary cells, immortal myogenic lines, including C2C12 (mouse) and L6 (rat) cells, are commonly used due to ease of culture and availability [384–387]. Yet, immortal cell lines exhibit low excitability [388] and poor physiological relevance compared to primary cells [389–391]. Induced pluripotent stem cells (iPSCs) are a promising alternative to traditional primary and immortal cultures due to their high expansion capability and potential sourcing from specific genetic backgrounds [382, 392–397]. BAM models have been used to examine physiological events such as hypertrophy and atrophy in response to drugs and exercise [398–401], skeletal muscle wounding and regeneration [400, 402, 403], force production [404–407], cell signaling [408–410], and drug response [411–414]. Importantly, as different muscle cell sources have distinct costs and benefits, different cell populations can be readily interchanged in BAM models to suit specific research needs.

Further advances have been made in the field of skeletal muscle tissue engineering through other approaches, such as scaffold free assemblies, bioprinting, and chip based systems. Scaffold free assemblies use the contractile nature of myotubes to form 3D tissues. In these systems, differentiated skeletal muscle/fibroblast monolayers delaminate from the culture substrate are rolled in on itself and pinned down to form “myoids” or “myooids” [378, 380, 415, 416]. Myoid models recapitulate many structural and functional features of native muscle, such as production of ECM, microvessels, and spontaneous contractions [417]. Although myoid constructs have been reported to be stable for up to 40 days, drawbacks include long maturation times (3–4 weeks), inability to scale cultures [418], and low force generation [401]. Recent advances in bioprinting technology have led to the printing of biomimetic muscle tissues and have been reviewed extensively [419, 420]. Bioprinting skeletal muscle is an appealing technique due to its high precision in cell positioning and alignment; however, progress in this area is limited by broad challenges in the field such as cell viability, printing speed, and resolution [419–422]. Additionally, printing the soft materials necessary to recapitulate the skeletal muscle microenvironment remains a challenge [423]. Recent “muscle-on-a-chip” devices have shown several advantages, including avoiding perfusion required to feed thicker tissues. Using microfabricated cultures, researchers have demonstrated muscle viability and enhanced maturation in response to microtopographical and morphological cues [424–426]. Skeletal muscle-on-a-chip systems are a promising tool for drug toxicity studies, especially due to their low media consumption and extensibility to high throughput screenings. Recently, a 3D skeletal muscle microdevice has been coupled with a biosensing platform to monitor myokine secretion. The authors validated this system by measuring IL-6 and TNF-α levels in response to electrical and biological stimulation [427]. However, muscle microdevices are limited by the need for specialized training and equipment to fabricate and use these devices.

It is important to emphasize that most of the models described above largely consist of homogeneous cell populations that lack the organization of native tissue. Recent progress has been made in incorporating heterogeneous cell population in BAMs, including the addition of endothelial cells and demonstration of vascular network formation [410, 417, 428–433]. In a mixed muscle/vascular mouse myoid model, researchers found high levels of vascular endothelial markers such as VEGF, CD31, and VE-cadherin, indicating the survival and signaling of vascular cells. Yet, the extent of the network formation was not examined in this study [417]. Endothelial vessel formation has been demonstrated on engineered skeletal muscle scaffold systems; however, muscle cells do not align along one axis, limiting contractility and tissue function [431]. Applying uniaxial strain to a vascularized mouse BAM model has been shown to induce vascular tube formation, likely through increased VEGF secretion by the differentiating muscle [433]. In a human vascularized BAM model researchers identified optimal cell seeding ratios (50–70% muscle cells) and media blends (endothelial growth media) for generating endothelial tubes along with aligned myofibers [429, 430]. Despite these advances, further work should be done to characterize vessel structure, and nutrient and oxygen delivery in vascularized BAMs. As a model of muscle regeneration, macrophages have been added into rat BAMs to study the regenerative potential of satellite cells within the engineered tissue. The incorporation of bone marrow derived macrophages showed recovered Ca^2+^ transients after injury compared to muscle only controls. Muscle-macrophage constructs also had improved cell organization and regeneration of myofibers post injury. Further, the authors demonstrate impaired regeneration in adult derived engineered muscle compared to neonatal constructs. In the future, this model can be used to identify pro-regenerative treatments in adult muscle [434]. Continued development of heterogeneous muscle models is of interest to the field of aging research given the prevalence of dysregulated adipose, fibroblast, and macrophage signaling with age.

BAMs have been used to study physiological muscle function, pharmaceutical response, and human disease [378, 412, 414, 435, 436]. While few systems have been developed in the context of aging (discussed below), other BAM models of disease demonstrate the power of the technique. Disease models of skeletal muscle include Miyoshi myopathy, Duchenne, limb-girdle, congenital muscular dystrophy, Pompe disease, and amyotrophic lateral sclerosis [437–445]. One strategy that is readily applicable is incorporating cells isolated from diseased patients into tissue constructs. As an example, Bersini and colleagues engineered myobundles co-cultured with endothelial cells and muscle-derived fibroblasts isolated from patients with Duchenne muscular dystrophy (DMD) [446]. Tissues with DMD fibroblasts exhibited an increased fibrotic phenotype characterized by higher collagen I and fibronectin deposition compared to healthy and TGF-β (inducer of fibrotic response) treated controls. Further, samples with DMD fibroblasts exhibited increases of α-smooth muscle actin compared to controls, indicating a shift towards a myofibroblast phenotype, consistent with the *in vivo* disease. The ability to capture and assay fibrosis, as demonstrated in the above models, has clear applicability to many aging studies.

In another study, human iPSCs from patients with DMD and limb-girdle muscular dystrophy were used to engineer 3D disease models with muscle, vascular, and neuronal cells [444]. These engineered muscles recapitulated disease phenotypes seen *in vivo* including the nuclear elongation typical in laminopathies. As another key example, BAMs generated from primary muscle cells isolated from both healthy individuals and patients with Pompe disease were used to test potential therapies [442]. Pompe disease myobundles exhibited traits consistent with that of clinical data such as elevated glycogen content and low acid alpha-glucosidase (GAA) gene activity. Researchers compared tissue functionality between healthy and Pompe disease models, observing reduced fatigue resistance, tetanic force production, and glycogen mobilization. While the observed functional defects were not alleviated by treatment with recombinant human GAA (current standard of care) or AAV-mediated GAA expression, the use of similar platforms for screening therapies is promising. Disease models such as the above can be readily adapted to study aging phenotypes by incorporating cell populations derived from aged individuals. The ability to compare functional and mechanical properties of aged and young muscle is of special interest to aging research, as elderly people have reduced muscle functionality. Further, being able to screen pharmaceutical interventions in muscle specific AAD models represents a significant advancement in the field of aging biology.

#### Tissue engineered muscle models to study aging

In recent years, engineered muscle has been used to study specific aging and aging associated diseases. A key example is the role muscle plays in insulin sensitivity and the age-related disease, type 2 diabetes (T2D). As aged muscle displays reduced insulin sensitivity [322, 447], it is especially relevant to quantify insulin sensitivity in engineered muscle. To test this, Kondash and colleagues created human myobundle constructs using primary myoblasts, differentiated in a 3D matrix for 2 weeks [436]. The authors found that 3D engineered constructs displayed a significantly higher glucose uptake in response to insulin than similarly cultured 2D cells. Further, the usefulness of this model for elucidating therapeutic mechanisms was also tested. Metformin, a common pharmaceutical for hyperglycemia and T2D, led to similar increases in glucose uptake in the presence or absence of insulin; indicating that metformin does not impact insulin responsiveness in peripheral muscle tissue. Further, metformin was found to impair both twitch and tetanus force production as well as decrease fatigue resistance. Although the magnitude of insulin response observed in this study is lower than that of native muscle tissue, the authors demonstrate the importance of the 3D microenvironment for improving physiological relevance in T2D studies. Additional work performed by Acosta and colleagues used engineered muscle to test the effect of systemic metabolic changes on muscle health [448]. Using muscle precursor cells isolated from lean, obese, and diabetic rats, engineered constructs were maintained in either myogenic media or adipogenic media. The authors showed that constructs with diabetic muscle precursor cells had decreased creatine kinase activity, tissue compaction, myotube alignment, and reduced tensile strength when compared to lean control samples. Overall, these data indicate diabetic myogenic precursor cells reduce overall muscle integrity. Further, the authors showed increased adipogenic differentiation in diabetic samples. Increased adipose presence between muscle fibers is common *in vivo* with aging, where muscle precursor cells are a potential source of adipose tissue [448]. These examples demonstrate tissue engineered skeletal muscle can be readily applied to the study of aging phenotypes such as increased insulin resistance and adipose infiltration.

In addition to the genetic and systemic factors discussed above, models of aged muscle have also been generated similar to the BAM method described above [449–451]. Sharples and colleagues utilized late passage C2C12 myoblasts to replicate aging phenotypes, including reduced myofiber diameter, length, and peak force development [449]. The reduced force generation observed coincides with a decrease in construct differentiation and hypertrophy potential. The authors quantified transcript expression of muscle differentiation and hypertrophy markers throughout culture. In aged constructs, they observed an increase in myostatin and TNFα, genes associated with impaired differentiation potential and sarcopenia [449]. A study performed by Rajabian and colleagues takes this work a step further by measuring calcium handling and metabolic function in aged human engineered muscle tissue [450]. Human myoblasts were obtained from young and aged donors and seeded into engineered constructs. Tissues formed from aged myoblasts exerted lower contraction force compared to younger control samples, fail to respond to electrical stimulation and, consistent with a lack of muscle contraction, have lower Ca^2+^ and ATP concentrations. Further, to study regeneration in aged tissue, the authors induced muscle injury using cobra cardiotoxin (CTX). Samples made with young myoblasts regenerated myofibers within 5 d post CTX injury, while aged constructs did not regenerate, resulting in reduced myotube diameter. Indeed, the number of multipotent satellite cells (identified with positive staining for PAX7) did not change after CTX injury in pre-senescent tissues, indicating increased regenerative potential [450]. Overall, these studies demonstrate that engineered skeletal muscle replicates many of the basic phenotypes seen with aging *in vivo*.

An additional application of engineered muscle is to elucidate the molecular mechanisms of aging. Shahini and colleagues leveraged engineered skeletal muscle to test the role of NANOG expression in mitigating senescence-associated dysfunction [451]. These studies were built off prior work showing NANOG expression reversed senescent phenotypes in MSC populations [452, 453]. In the skeletal muscle study, late passage C2C12 myoblasts were engineered to express NANOG under the control of tetracycline and embedded in a 3D collagen/Matrigel matrix. The authors observed NANOG expression partially rescued myotube population levels, diameter, and length to that of early passage controls when compared to late passage constructs without NANOG. They further observed a restoration of differentiation markers MYHC and Actinin. A key advantage of engineered muscle models, demonstrated by the above studies, is the accessibility for targeted genetic and pharmacological manipulation. As with other models, the advantages of engineered muscle cultures are coupled to limitations, discussed below.

#### Limitations

As with other organotypic models, exclusion of cell types present *in vivo* is a challenge for skeletal muscle as well. For example, common aging phenotypes of inflammation, reduced peripheral vascularization, and adipose infiltration require inclusion of immune cells, endothelial cells, and adipocytes. In addition to sourcing and maintaining these cells, co-culture with muscle cells presents additional challenges due to their high metabolic demand and contractility. Progress is being made, for example with inclusion of increasingly complex vascular components [410, 417, 428–433], but there are many areas needing improvement.

Further, skeletal muscle poses unique challenges for cell sourcing. Most *in vitro* models of aging skeletal muscle are established from primary cells that are derived from animal models and patients [450, 454–456]. Although primary cells offer increased physiological relevance relative to immortalized lines, the culture methods needed to isolate and expand these cells to populations suitable for organotypic studies rely on specialized techniques and restricted supplies, especially for human cells. Established cell lines are a more accessible sourced of aged myoblasts, and replicative senescence models have been established and used in 3D culture [449]. While the tradeoffs between primary cells and established cell lines are well documented for any *in vitro* culture system, the large number of cells needed for organotypic skeletal muscle models can make sourcing sufficient primary tissue difficult.

It is important to note that skeletal muscle is typically composed of multiple fiber types, with different physiology and function. In aging, fast twitch fibers preferentially atrophy, leading to changes in fiber composition. While an important phenotype, especially in aging, fiber type is typically not assessed or controlled in organotypic models, leading to an important capability gap [457]. Further, engineered skeletal muscle generates force several orders of magnitude lower than that of adult human muscle, with reduced myofiber diameters [458]. Methods to improve contractile properties in these models focus on co-culture with motor neurons, electrical and mechanical stimulation, and improved nutrient and gas delivery. Ultimately, better control of muscle differentiation and maturation will improve modeling of both healthy and aged tissues.

Finally, although both males and females exhibit loss of muscle mass with age, the pattern of decline is sex dependent. Similar to other tissues, organotypic constructs could be ideal platforms to isolate the impact of sex specific cells and specific hormone levels on muscle function [459, 460]; however, fully capturing the systemic sex differences *in vitro* is beyond the current capabilities of these models.

## Discussion and outlook

Progress in tissue engineering has resulted in the development of three-dimensional organotypic models, and these have demonstrated potential to overcome several limitations of current aging models. Organotypic models, while not replacing animal models, have multiple advantages, including lower cost, increased accessibility, and human-specific biology. This allows for re-capitulation of human disease and aging phenotypes that animals may not experience naturally or may experience differently [7, 102]. Further, tissue engineered organotypic models have advantages over classic two-dimensional *in vitro* models as they incorporate physiologically important structural-cell and cell-cell interactions [71]. Additionally, tissue engineered cultures offer flexible scalability when compared to organoid and microchip culture formats. Appropriately scaled models are especially important when investigating aging; in many cases, aging contributes to breakdown of disruption and alterations of the overall tissue, and may include altered nutrient diffusion, organization, and cell-cell communication. In addition, tissue engineered models offer high customizability compared to conventional *in vivo* models, where specific cell populations or biomaterials can be easily selected or replaced to match research needs. In the three tissues that were addressed here, we highlighted studies that have specifically adapted these models to studying aging; where possible we have also highlighted the accessibility of these models to research groups that may not have prior experience. Importantly, organotypic models are straightforward to customize and, with some optimization, can be a reliable and powerful tool for any aging researcher to adapt to their needs and questions.
